# Inflammasome Signaling: A Novel Paradigm of Hub Platform in Innate Immunity for Cancer Immunology and Immunotherapy

**DOI:** 10.3389/fimmu.2021.710110

**Published:** 2021-08-05

**Authors:** Ying Li, Jiao Lv, Weikai Shi, Jia Feng, Mingxi Liu, Shenao Gan, Hongjin Wu, Weiwei Fan, Ming Shi

**Affiliations:** ^1^School of Life Science and Technology, Harbin Institute of Technology, Harbin, China; ^2^International Research Center for Regenerative Medicine, BOAO International Hospital, Qionghai, China; ^3^Department of Infectious and Medicine, Heilongjiang Provincial Hospital, Harbin, China

**Keywords:** inflammasome, pyroptosis, NETosis, innate immunity, gasdermins, cancer immunotherapy

## Abstract

Inflammasomes are fundamental innate immune mechanisms that promote inflammation and induce an inflammatory form of programmed cell death, pyroptosis. Pyroptotic inflammasome has been reported to be closely associated with tumorigenesis and prognosis of multiple cancers. Emerging studies show that the inflammasome assembly into a higher-order supramolecular complex has been utilized to evaluate the status of the innate immune response. The inflammasomes are now regarded as cellular signaling hubs of the innate immunity that drive the production of inflammatory cytokines and consequent recruitment of immune cells to the tumor sites. Herein, we provided an overview of molecular characteristics and biological properties of canonical and non-canonical inflammasome signaling in cancer immunology and immunotherapy. We also focus on the mechanism of regulating pyroptotic inflammasome in tumor cells, as well as the potential roles of inflammasome-mediated pyroptotic cell death in cancers, to explore the potential diagnostic and therapeutic markers contributing to the prevention and treatment of cancers.

## Introduction

Inflammasome, a major class of signalosomes in innate immunity, is a cytosolic multiprotein platform that formed by the oligomerization of a sensor, an adaptor apoptosis-associated speck-like (ASC) and caspases in response to pathogen-associated molecules and cellular stress (1, 2). These inflammasome components are expressed at low levels in normal tissue cells to prevent inappropriate activation, and are primed, activated and assembled through homotypical death domain (DD) interactions (3–6). The DD domains, from caspase recruitment domain (CARD), pyrin domain (PYD), to death effector domain (DED), were found to self-assemble into higher-order helical filaments in inflammasome (1). The higher-order inflammasome complexes carry out intricate signaling and key effector functions in innate immunity and inflammation.

Inflammasome activation induces pyroptosis, a type of programmed cell death. Studies have shown that the gasdermin family members (Gasdermins, GSDMs) play vital roles in inflammasome-induced pyroptosis (7–9). The inflammasome-induced pyroptosis depends on the formation of plasma membrane pores by the pyroptosis effectors GSDMs (7). Emerging studies had indicated that inflammasome activation plays a central role in the tumorigenesis (including immunosuppression, proliferation, angiogenesis and metastasis) and tumor suppression (10). The pyroptosis initiated by inflammasomes induces innate immune responses in cancer tissues, and targeting pyroptosis has exhibited potential anti-tumor capabilities in cancer treatment (8). Thus, targeting the inflammasome and pyroptosis is a promising strategy for cancer immunotherapy. The inflammasome and its related pyroptosis-trigged immune activation in cancer tissues will provide cancer patients with more effective anti-tumor immune responses and a better prognosis (9).

Activation of cell death effector GSDMs also has some connections with a type of cell apoptosis, NETosis, which is related to the formation of inflammasome and noncanonical inflammasome signaling (11, 12). In previous studies, NETosis was believed to associate with the immune defenses, helping to resist various pathogens (13, 14). While emerging studies have shown that noncanonical inflammasome signaling-elicited NETosis also has a positive impact on the tumorigenesis by protecting tumor cells against immune attack and promoting tumor cell metastasis (15–17).

Currently, with an increasing number of studies in innate immunity, the inflammasome assembly into a functional higher-order complex functions as hub platforms for inflammatory cytokine production, and has been considered to utilize in evaluating the activation and regulation status of the innate immune response (2, 18, 19). The inflammasomes are now regarded as cellular signaling hubs of the innate immunity that drive the inflammatory signaling and consequent recruitment of immune cells to the tumor sites, but activation of different inflammasomes may exhibit the exact opposite outcomes in cancers, anti-tumor or pro-tumor effects (20–23). In-depth understanding the functions of these canonical and non-canonical inflammasomes is critical for revealing the molecular mechanisms that govern the innate immune response and inflammatory signaling in cancer Immunotherapy (10, 24).

## Canonical and Non-Canonical Inflammasome Signaling in Tumor Immunity

Canonical inflammasomes, assembled by sensor proteins (including pyrin domain containing related protein family (NLRP), absent in melanoma (AIM) 2, interferon-γ inducible factor (IFI) 16, RIG-I, and CARD-domain-containing (NLRC) 4), play key roles in immune surveillance of pathogens infections and danger signals by proteolytically activating caspases 1 and/or 11 (caspase-4/5 in humans) that cleaves interleukin-1β (IL-1β), interleukin-18 (IL-18) and the pore-forming protein gasdermin D (GSDMD), leading to cytokine maturation and pyroptosis (**Table 1**) (25, 26). Canonical inflammasome-induced pyroptosis is typically marked by the induction of rapid polymerization of the bipartite adapter ASC into large helical filaments with the sensors and caspases to form a single supramolecular ASC punctum (also known as ASC specks), which mediates robust cellular responses and acts as an important hallmark for inflammasome activation (27–29). NAIPs, which could recognize the bacterial ligands, recruit NLRC4 to assembly the NAIP-NLRC4 inflammasome complex, and directly activate the caspase 1 without the adaptor ASC (30–32).

**Table 1:** Canonical and non-canonical Inflammasomes.

	Sensor proteins	Adaptor - Caspase	Activated caspases	Cleaved proteins	Cytokine release	Cell death
Canonical inflammasomes	NLRP family	adaptor protein ASC - caspase-1	caspase-1	GSDMD, pro-IL-1β, pro-IL-18	IL-1β, IL-18	pyroptosis; apoptosis;
	AIM2					
	IFI16					
	RIG-I					
	NAIP-NLRC4	caspase-1				
Non-canonical Inflammasomes	NLRP family	caspase-11	caspase-1, 11	GSDMD, pro-IL-1α, pro-IL-1β, pro-IL-18	IL-1α, IL-1β, IL-18	pyroptosis; apoptosis; necroptosis
	ZBP1 dependent - NLRP3	caspase-8, 6				
	Inhibition of TAK1 or IkappaB kinase (IKK)	caspase-8				
		TAK1 dependent - caspase-8	caspase-8			

**Figure 1:**
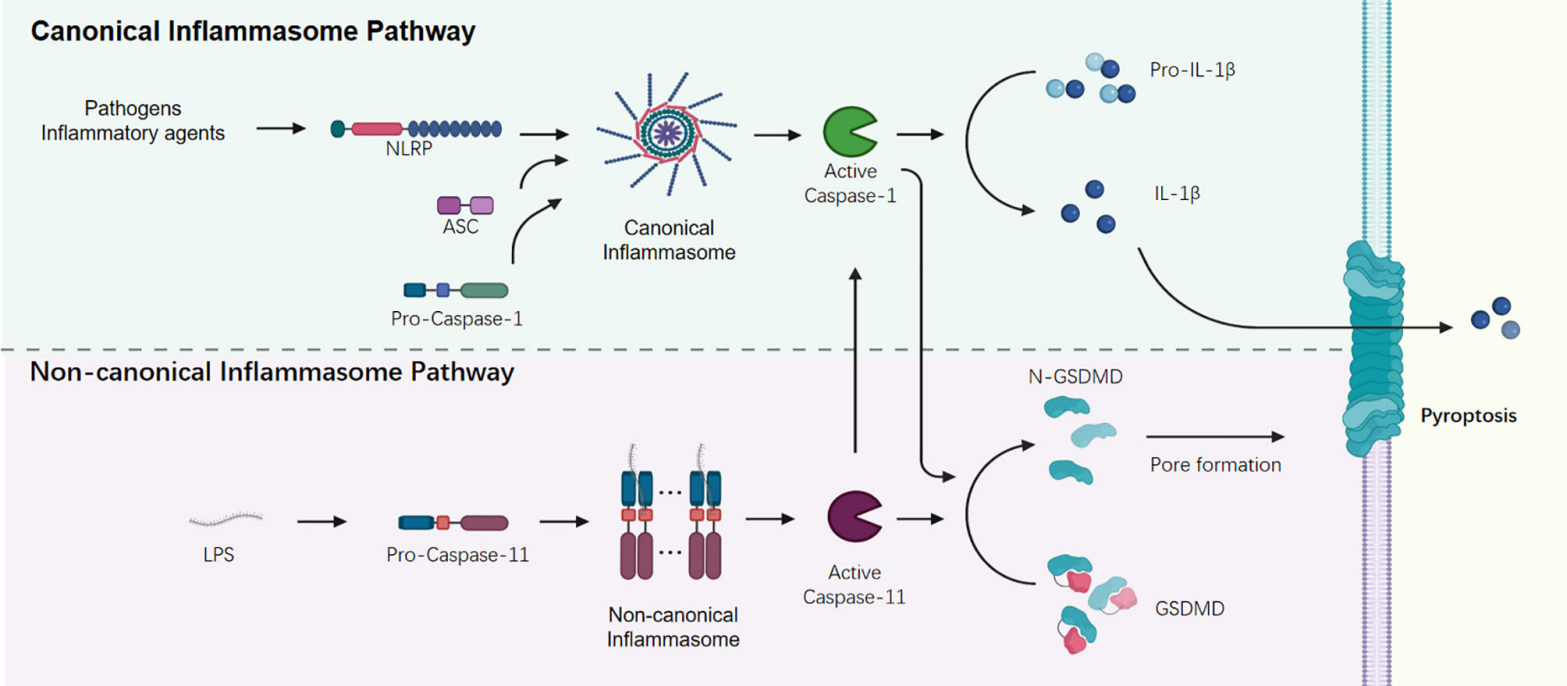
Canonical and non-canonical Inflammasome signaling pathways.

The non-canonical inflammasome activation mediates a caspase 11 (caspase 4/5 in human) dependent innate immune response to the invasion of gram-negative bacteria (33–35). The cytosolic sensor caspase 11 functions as a signal initiator and mediates the recognition of gram-negative bacteria *via* directly interacting with cytosolic lipopolysaccharide (LPS) and assembling a higher order structure called the non-canonical caspase 11 inflammasome (33). Besides caspase 11, innate immune sensor ZBP1 and the inhibition of kinase TAK1 could regulate the assembly of RIPK1/RIPK3-FADD-caspase-8 cell death complex and induce the Pyroptosis, Apoptosis, and Necroptosis (PAN-optosis) (**Table 1**) (36, 37). These active caspases cleave and activate GSDMD to promote pyroptosis, and then trigger a secondary activation of the canonical NLRP3 inflammasome for cytokine release (**Figure 1**) (38, 39).

The effects of both canonical and non-canonical Inflammasomes activation-induced pyroptosis may be a double-edged sword on cancers (40). The role of inflammasome activation in promoting tumorigenesis has been previously reviewed by rajendra karki et al., which indicates that inflammasome components could induce cancer cell proliferation, survival and metastasis, and promote cancer cells to evade immune surveillance (10). Except the direct killing of cancer cells and cancer related microenvironmental cells by pyroptosis, the release of inflammasome-dependent cytokines (IL-1β, IL-18, et al.) and other costimulatory molecules (either from the cancer cells or from the cells in the tumor microenvironment) will significantly reshape the cancer immune microenvironment. The composition of cancer immune microenvironment will determine the effect of pyroptosis on cancer. On the one hand, pyroptosis may promote the cancer occurrence by recruiting the immunosuppressive immune cells (such as myeloid-derived suppressor cells, MDSC) (41–43) and inducing chronic inflammation (10, 44, 45); on the other hand, it may also inhibit the cancer occurrence by recruiting the NK and CD8+ T cells to the cancer microenvironment (8).

## GSDMs and GSDMs-Dependent Canonical Inflammasome Signaling Modulate Tumor Immunity

The GSDMs, pyroptosis executors, are consists of gasdermin A (GSDMA), gasdermin B (GSDMB), gasdermin C (GSDMC), gasdermin D (GSDMD), gasdermin E (GSDME), and Autosomal Recessive Deafness Type 59 Protein (DFNB59 or PJVK) in homo sapiens, and displayed different tissue expression patterns (46, 47). In 2015, Kayagaki, N. et al. (38), Shi, J. et al. (48) and He, W. T. et al. (49) firstly discovered GSDMD, the executor of pyroptosis, and confirmed that it was cleaved by caspase 1 and caspase 4. Since this discovery, more and more gasdermins were characterized to play vital roles in inflammasome and pyroptosis. Further protein structure analysis of these GSDMs confirmed that by cleaving and releasing their N-terminal domains, these GSDMs can induce cell death by forming large oligomeric pores on cell membrane, disrupting the integrity of cell membrane and releasing the inflammatory mediators (50–54). These functions and mechanisms recently had been reported to relate with cancer therapy, especially the GSDMD and GSDME (8, 55, 56).

GSDMA, GSDMC and PJVK are not detected in most human tissues (both tumor tissues and normal tissues), while GSDMB, GSDMD and GSDME are highly expressed in most human tissues (both tumor tissues and normal tissues), especially GSDMD (47). These also indicate that different GSDMs may perform different functions in cancer development and cancer therapy (**Table 2**).

**Table 2:** Function of GSDMs in anti-tumor immunotherapy.

GSDMs	Upstream Effector	Activated Caspase	Effector cells	Target Cells	Cell death	Reference
**GSDMA3**	—	—	Immune cells	GSDMA3^+^ tumor cells		(52, 55)
**GSDMB**	Granzyme A (Directly cleave GSDMB)	—	NK-92MI/CAR-T/TCR-T cells/CTLs	GSDMB^+^ cells		(57)
**GSDMC**	TNFα	Caspase-8	Macrophages	Cancer cells		(58)
**GSDMD**	Bacterial Endotoxin - Lipopolysaccharide (LPS)	Caspase-11	Gram-negative bacteria	Macrophages, Endothelial cells	pyroptosis	(38, 59–61)
	bacterial lipopolysaccharide	Caspase-1 and Caspase-4/5/11	—	mouse bone marrow Macrophages	inflammasome-activated caspase-1 and LPS-activated caspase-11/4/5	(48, 51, 54)
	The inhibition of TAK1 or IkappaB kinase (IKK) by the Yersinia effector protein YopJ	Caspase-8/RIPK1	—	Macrophages	NLRP3 inflammasome-dependent release of interleukin-1beta (IL-1beta)	(37)
	intracellular protease inhibitors Serpinb1a and Serpinb6a	Cathepsin G (CatG)	—	Monocyte and Neutrophil		(62)
	GSDMB	Caspase-4	—	Leukocytes	non-canonical pyroptosis	(63)
	Liver injury	Caspase-8	—	Hepatocyte		(64)
	—	Caspase-4/11	—	Hepatocyte		(65)
	—	FADD and Caspase-8	—	intestinal epithelial cell (IEC)	MLKL-induced necroptosis and caspase-8-GSDMD-dependent pyroptosis-like death	(66)
	AIM2 inflammasome	Caspase-1	—	HEK293 cells		(67, 68)
	NLRP3 inflammasome	Caspase-1	—	Neutrophils		(69)
**GSDME**	Bid-caspase-9-caspase-3 axis	Caspase-1	—	GSDMD-low/null cell types		(70)
	BRAFi + MEKi	Caspase 3	—	—		(9)
	chemotherapy drugs or TNFα	Caspase-3	—	GSDME^+^ tumor/primary cells		(56, 71–74)
	Granzyme B	Caspase 3	CAR-T cells	GSDME^+^ leukemic/target cells		(75)
	Granzyme B	Caspase 3	NK and CD8^+^ T lymphocytes	GSDME^+^ tumor cells		(8)

GSDMA, especially GSDMA3, is expressed in the epidermis and frequently silenced in gastric cancer cell lines (76). Mutations in GSDMA3 with gain-of-function are associated with skin inflammation and hair loss (77). GSDMA3-N domains could form membrane-disrupting pores during pyroptosis (50). Dysregulated GSDMA3 could cause cell necrosis and chronic inflammation (52), and potentially influence the cancer immunotherapy (55).

**Figure 2:**
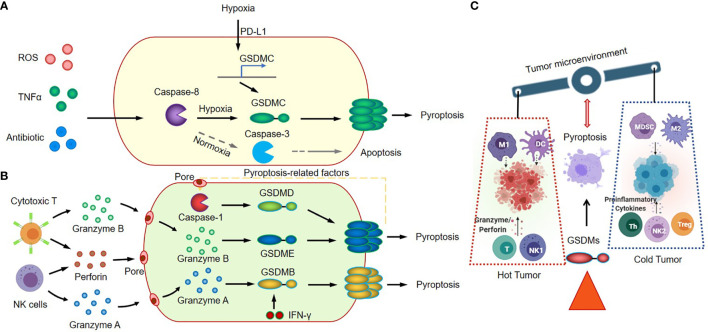
Canonical inflammasome signaling in cancer immunology. **(A)** GSDMC-dependent inflammasome signaling and pyroptosis pathway. **(B)** Granzyme A/B-mediated cytotoxic lymphocyte killing mechanism and GSDMB/GSDMD/GSDME induce tumor cell pyroptosis. **(C)** GSDMs-mediated inflammasome activation and pyroptosis regulate antitumor immunity.

GSDMB, which has been proven as an independent poor prognostic biomarker in breast cancer, is overexpressed in about 60% of HER2 breast cancers (78). The highly expressed GSDMB significantly promotes cancer cell migration and develops cancer cell resistance to anti-HER2 therapies (79). Knockout GSDMB, or intracellular-delivered anti-GSDMB through nanocapsules could neutralize the effects of GSDMB, reduce the aggressiveness of HER2 breast cancer, and enhance the sensitivity to trastuzumab (80). These results indicate that GSDMB may play a positive role in cancer development. However, Zhou Z et al. found that GSDMB positive cells showed greater sensitivity to granzyme A-mediated cytotoxic lymphocyte killing mechanism, and the upregulation of GSDMB expression in tumor cells might be due to the activation of the interferon gamma signaling (57) (**Figure 2B**). While the interferon gamma signaling also could trigger the expression of immune checkpoints, such as PD-L1 and PD-L2 (81, 82), which provided the explanation for the poor prognosis of GSDMB-positive tumors. In addition, these studies also indicated that cancer immunotherapy with the immune checkpoint inhibitors might be a better strategy for treating the GSDMB-positive tumors. We are looking forward to see the similar clinical trials for this cancer subtype in the future (83).

GSDMC, another effector of pyroptosis, correlates with poor survival in cancer patients (84, 85). GSDMC is cleaved by caspase-8 with TNFα treatment, and also can be cleaved by caspase-6 in response to reactive oxygen species (ROS) insult in cancer cells. The elevated expression level of GSDMC, which could be induced by the nuclear translocated PD-L1, is required for the switching TNFα-induced apoptosis to pyroptosis in cancer cells (58). In tumor microenvironment, the tumor-associated macrophages could secret the TNFα, and induce tumor necrosis through the activation of caspase-8, the translocation of PD-L1, and the cleavage of GSDMC (**Figure 2A**). This GSDMC-dependent inflammasome signaling and pyroptosis pathway will significantly change the tumor microenvironment, promote tumor progression and increase the resistance to chemotherapy, radiotherapy and immunotherapy.

The functions of GSDMD and GSDME are much clearer (48, 51, 54, 59–61). Studies had showed that GSDMD and GSDME-mediated canonical inflammasome signaling and pyroptosis play vital roles in the immune response of cancer tissues through modulating the tumor immune microenvironment. The GSDMD could be cleaved by activated caspase 4/11 (63, 65) and caspase 8 (64, 66), and activated caspase 1 (activated by AIM2 or NLRP3 inflammasome) (67–69). The GSDME could be cleaved by activated caspase 1 and caspase 3, and then trigger the transition of cancer cells from apoptosis to pyroptosis (9, 70, 86). The transition highly relies on the expression level of GSDME in cancer cells (56). The cleavage of GSDMD and GSDME in cancer cells could be induced by various therapeutic strategies, including chemotherapy drugs (56, 71–74, 87), molecular target therapies (62, 88), or immune cell therapy (8, 75). The GSDME is constitutively expressed in many normal tissues, which explains why the chemotherapy drugs could induce direct damage in normal tissues (56). Remarkably, these damages in tumor tissues have a positive function; the damage-induced cleavage of gasdermins, inflammasome activation and subsequent pyroptosis will promote the recruitment and activation of the tumor-infiltrating NK and CD8+ T lymphocytes in tumor sites (8). Thus, GSDMs-mediated inflammasome activation and pyroptosis could turn “cold” tumor into “hot” by modulating tumor immune microenvironment, and consequently regulate antitumor immunity (**Figure 2C**).

The expression pattern of GSDME is distinguished from GSDMD. The GSDME gene is frequently silenced in cancer cells, and loss of function (LOF) of GSDME by mutations or hypermethylation of promoter region in cancer cells will significantly reduce the anti-tumor innate immune responses (8, 40). Zhang Z et al. reported that the granzyme B released by cytotoxic T cells could cleave GSDME in cancer cells, and the granzyme B/GSDME-mediated pyroptosis suppressed tumor growth through a perforin-dependent T-cell killing mechanism (8). Thus, it will be beneficial to target GSDME or to elevate tumor-derived GSDME expression level in cancer treatment. While the excessive activation of inflammasome-induced pyroptosis in cancer treatment, such as in CAR-T therapy, could also cause serious consequences; The CAR-T cells elicited GSDME–mediated cancer cell pyroptosis and released pyroptosis-related factors. The pyroptosis-related factors activated caspase 1 for GSDMD cleavage in macrophages, and resulted in the release of more cytokines and the subsequent cytokine release syndrome (CRS) (75) (**Figure 2B**).

## Noncanonical Inflammasome Signaling-Elicited NETosis Promotes Tumorigenesis

**Figure 3:**
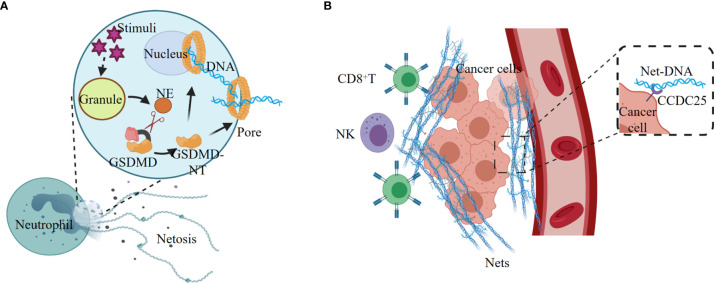
NETosis elicited by noncanonical inflammasome signaling promotes tumorigenesis. **(A)** Various stimuli promote the release of NE from the granules, and NE cleaves and activates GSDMD, leading to nuclear and plasma membrane rupture and neutrophil cell lysis by NETosis. **(B)** Tumor-secreted ligands induce extrusion of NETs, and NETs protect tumor cells from CTL and NK cytotoxicity. The extracellular NETs-DNA binds to the transmembrane protein CCDC25 on tumor cells, and thus improve tumor cell migration.

NETosis, a proinflammatory cell death modality originally identified in neutrophil, provides host defense against extracellular intruders in response to various stimuli. NETosis differs from apoptosis and necrosis, but has some connections with the activation of pyroptosis executor GSDMD (12). Various stimuli promote the release of NE from the neutrophil granules, and NE cleaves and activates GSDMD, leading to nuclear and plasma membrane rupture and neutrophil cell lysis by NETosis (**Figure 3A**). Exposure of neutrophil to cytosolic LPS also activates the noncanonical inflammasome signaling and triggers GSDMD-dependent NETosis (12, 89). Both Caspase-11 and GSDMD are required for NETosis at multiple stages, including nuclear delobulation, chromatin decondensation, nuclear membrane permeabilization and plasma membrane rupture (89).

Neutrophil extracellular traps (NETs) are the regulated outcome of NETosis, and the release of NETs is linked with tumorigenesis (90–92). Tumor cells can recruit myeloid cells, mostly neutrophils, by secreting CXCR1 and CXCR2 agonists ELR positive CXCL chemokines, such as CXCL1, CXCL2, and CXCL8 (93). The tumor-derived ELR positive CXCL chemokines are the major mediators of cancer-promoted NETosis and NETs (91). The NETs released from neutrophils are enriched on the tumor surface to form a barrier, which effectively reduces the contact of CD8+ T cells and NK cells with tumor cells, and thus protects tumors from immune cytotoxicity (**Figure 3B**).

Excessive NETs produced by sustained inflammation contribute to reawakenment of dormant cancer cells (94). The sustained lung inflammation induced by tobacco smoke or LPS instillation recruits and activates neutrophils, and the subsequent NETs formation is greatly induced in the cancer cell dormancy mouse model. The two NETs-associated proteases neutrophil elastase (NE) and matrix metalloproteinase 9 (MMP9) remodel the extracellular matrix (ECM) by hydrolyzing laminin. The proteolytic remodeling laminin induces the proliferation of dormant cancer cells through activating the cell surface ECM receptor integrin α3β1 (94). In mouse models of small bowel tumors, tumor development is related with the accumulation of low-density neutrophils (LDNs). The LDNs aggregation-induced up-regulation of complement 3a receptor (C3aR) and activation of the complement cascade lead to NETosis, coagulation and differentiation of neutrophils into N2 type, which in turn promote tumorigenesis (95).

NETs also play an important role in promoting tumor metastasis. The transmembrane protein CCDC25 is a NET-DNA (the DNA component of NETs) receptor on cancer cells, which activate the integrin‐linked kinase (ILK)/β-parvin pathway by binding to extracellular DNA to enhance the mobility of cells, thereby promoting tumor metastasis (96) (**Figure 3B**). In tissues of lung and retina, NETosis can induce apoptosis of senescent vascular endothelial cells and promote the formation of new blood vessels that are conducive to tumor growth (97, 98). In addition, NETs can continuously deposit in the lungs, which might be the reason why lung is one of the most common sites of cancer metastasis (99).

## Pyroptotic Inflammasome Signaling Acts as a Critical Regulator of Inflammation and TILs Within Tumor Microenvironment

The activation of canonical or non-canonical inflammasome signaling in the cytosolic compartment will lead to pyroptosis (34, 100, 101), which are critical defense mechanisms against endogenous (tissue or cellular injury) or exogenous danger signals (infections, such as microbes) (34, 100–102). The dysregulation of inflammasome activation in cancer development and progression is controversial, due to the inconsistent findings on potential cancer promotion and immunotherapy (103). As the potent contributors to the activation of inflammatory cytokines in cancer tissues, the excessive inflammasome signaling will lead to the cancer progression (10, 104, 105). Thus, inhibition of inflammasome with some certain inhibitors could potentially be used for clinical cancer treatment (106, 107). Meanwhile, in the treatment of cancer, various drugs could induce the activation of inflammasome-related pyroptosis and cause the release of proinflammatory cytokines. Subsequently, the activated inflammatory cytokines could recruit the NK or cytotoxic T cells to the tumor site for killing cancer cells, and eventually delay the tumor progression (108, 109).

Regulation of inflammasome activation might reinforce anti-tumor immunity by boosting the recruitment of TILs (110). Mechanismly, the checkpoint molecule, T cell immunoglobulin and mucin-containing molecule 3 (TIM-3) in DC cells restrains anti-tumor immunity through suppressing inflammasome activation; TIM-3-deficient DCs promote the recruitment of stem-like CD8+ TILs and boost antigen-specific immunity *via* increasing accumulation of reactive oxygen species resulting in driving inflammasome activation (111). Additionally, the pyroptotic inflammasome-cytokine (IL-18) pathway effectively regulates the NK-cell-mediated tumor attack through promoting the maturation of NK cells and surface expression of the death ligand FasL, which consequently leads to elevate the tumoricidal activity of NK cells (108). Thus, inhibition of inflammasome activation, or downstream effector cytokines might abrogate the protective anti-tumor immunity and expanded TILs.

**Figure 4:**
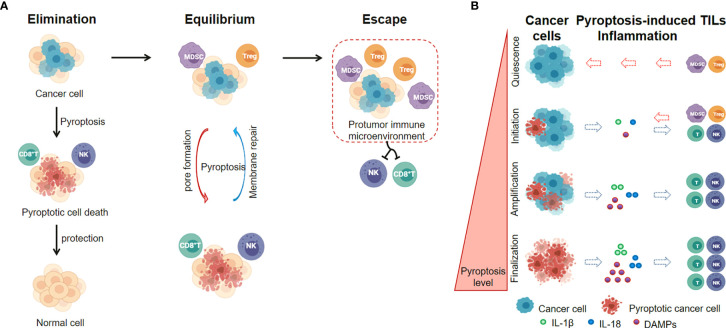
Pyroptotic Inflammasome signaling regulates inflammation and TILs within tumor microenvironment. **(A)** The level of pyroptotic inflammasomes is associated with the balance between cancer cell elimination and immune escape. **(B)** Pyroptosis levels in cancer cells affect the inflammation and TILs within tumor microenvironment.

Recently, emerging insights in cancer immunology indicate that the roles of pyroptotic inflammasomes on tumor immunotherapies may highly rely on the tumor stage and the tumor microenvironment (105). In early-stages of cancer development, the pyroptotic inflammasomes participate in the innate immune response, recruit tumor-infiltrating lymphocytes and promote inflammatory cell death of cancer. The immune cells recruited to tumor sites retain their immunosurveillance properties and anti-tumor immunity prevails (Elimination phase) (112), while the ESCRT-III-mediated plasma membrane repair in pyroptotic cells strongly inhibits pyroptotic cell death (113, 114). A dynamic interplay of pyroptosis with ESCRT-mediated membrane repair in cancer cells occurs in immune equilibrium phase. In addition, cancer cells also develop a series of pyroptosis-resistance strategies to escape immune attack and establish a protumor immune microenvironment (escape phase) (**Figure 4A**). The levels of pyroptotic inflammasomes regulate the inflammation and TILs within tumor microenvironment, and affect the balance between cancer cell elimination and immune escape (**Figure 4B**).

The inflammasomes have been considered as cellular signaling hubs of the innate immunity that drive the production of inflammatory cytokines, promote inflammation and induce pyroptosis in cancer cells. While inflammasome signaling hubs function in innate immune response, the inflammasome activation links with diverse physiological and pathological processes, such as autophagy (2, 115), cellular stress response (116), cell-cycle progression (117). In these processes, inflammasomes activation is tightly regulated by DDX3X-mediated assembly of stress granules, HDAC6-associated autophagosomal degradation, and NEK7-dependent mitotic spindle formation and cytokinesis (2, 115–117).

## Conclusion

Inflammasome signaling has shifted the paradigm for the hub platform in innate immune responses. The inflammasomes are considered as cellular signaling hubs of the innate immunity that drive the production of inflammatory cytokines, promote inflammation and induce pyroptosis in cancer cells. The polymerization of pattern recognition receptors, adaptor ASC, and effectors caspases into higher-order supramolecular complexes facilitates signal transduction cascades and proximity-facilitated enzyme activation. In these complexes, pattern recognition receptors (sensor proteins) and adaptor ASC form the center (ASC specks), whereas caspases make up the filaments. The inflammasome activation and assembly into higher-order supramolecular complexes function as inflammasome hub platforms for inflammatory cytokine production, and have been utilized to evaluate the status of the innate immune response.

The pyroptotic inflammasome regulates inflammation, TILs within tumor microenvironment, and the consequent recruitment of immune cells to the tumor sites. But the ESCRT-III-mediated plasma membrane repair in pyroptotic cells strongly inhibits pyroptosis in cancer cells. Thus, a dynamic interplay of pyroptosis with membrane repair in cancer cells occurs in immune equilibrium phase. The level of pyroptotic inflammasomes might related with the balance between cancer cell elimination and immune escape. With the inflammasome examples in cancer immunology presented here, we could see that the regulation of inflammasome level with some certain agonists or antagonists would potentially be used for future clinical cancer treatment.

## Author Contributions

All authors listed have made a substantial, direct, and intellectual contribution to the work, and approved it for publication.

## Conflict of Interest

The authors declare that the research was conducted in the absence of any commercial or financial relationships that could be construed as a potential conflict of interest.

## Publisher’s Note

All claims expressed in this article are solely those of the authors and do not necessarily represent those of their affiliated organizations, or those of the publisher, the editors and the reviewers. Any product that may be evaluated in this article, or claim that may be made by its manufacturer, is not guaranteed or endorsed by the publisher.
